# 3-Benzyl-5-benzyl­idene-2-sulfanylidene-1,3-thia­zolidin-4-one

**DOI:** 10.1107/S1600536811027450

**Published:** 2011-07-23

**Authors:** Durre Shahwar, Muhammad Asam Raza, Saherish Aslam, Sumbal Mehmood, Sidra Tariq, Abdullah M. Asiri

**Affiliations:** a Department of Chemistry, Government Collge University, Lahore 54000, Pakistan; b The Center of Excellence for Advanced Materials Research, King Abdul Aziz University, Jeddah, PO Box 80203, Saudi Arabia

## Abstract

In the title mol­ecule, C_17_H_13_NOS_2_, the essentially planar thia­zole ring (r.m.s deviation 0.005 Å) forms dihedral angles of 16.85 (8)° and 75.02 (8)° with the phenyl rings. The dihedral angle between the two phenyl rings is 61.95 (9)°.

## Related literature

For the synthesis and related structures, see: Shahwar *et al.* (2009 ▶, 2011 ▶).
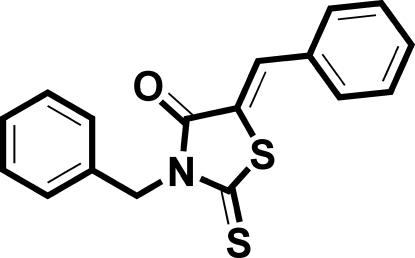


## Experimental

### 

#### Crystal data

C_17_H_13_NOS_2_
*M* *_r_* = 311.40
Triclinic, 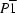

*a* = 6.3152 (2) Å
*b* = 10.8168 (3) Å
*c* = 11.4545 (3) Å
α = 84.1150 (9)°
β = 77.6000 (9)°
γ = 76.1770 (9)°
*V* = 740.99 (4) Å^3^
*Z* = 2
Mo *K*α radiation
μ = 0.36 mm^−1^
*T* = 296 K
0.35 × 0.31 × 0.15 mm

#### Data collection

Bruker Kappa APEX II CCD diffractometer
Absorption correction: multi-scan (*SADABS*; Bruker, 2007 ▶) *T*_min_ = 0.886, *T*_max_ = 0.949
13205 measured reflections
3583 independent reflections
2930 reflections with *I* > 2σ(*I*)
*R*_int_ = 0.028

#### Refinement

*R*[*F* ^2^ > 2σ(*F* ^2^)] = 0.036
*wR*(*F* ^2^) = 0.101
*S* = 1.03
3583 reflections
190 parameters
H-atom parameters constrained
Δρ_max_ = 0.28 e Å^−3^
Δρ_min_ = −0.26 e Å^−3^

### 

Data collection: *APEX2* (Bruker, 2007 ▶); cell refinement: *SAINT* (Bruker, 2007 ▶); data reduction: *SAINT*; program(s) used to solve structure: *SHELXS97* (Sheldrick, 2008 ▶); program(s) used to refine structure: *SHELXL97* (Sheldrick, 2008 ▶); molecular graphics: *PLATON* (Spek, 2009 ▶); software used to prepare material for publication: *WinGX* (Farrugia, 1999 ▶) and *PLATON*.

## Supplementary Material

Crystal structure: contains datablock(s) I, global. DOI: 10.1107/S1600536811027450/lh5281sup1.cif

Structure factors: contains datablock(s) I. DOI: 10.1107/S1600536811027450/lh5281Isup2.hkl

Supplementary material file. DOI: 10.1107/S1600536811027450/lh5281Isup3.cml

Additional supplementary materials:  crystallographic information; 3D view; checkCIF report

## Figures and Tables

**Fig. 1.**
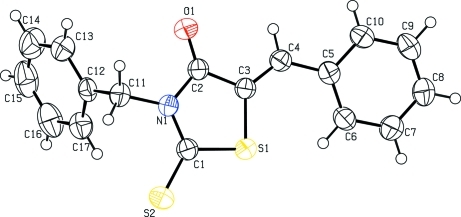
The molecular structure of (I) with thermal ellipsoids drawn at the 50% probability level.

## supplementary crystallographic information

### Comment

The crystal structure determination of the title compound (I) is a countinuation of our work on thiazolidinone derivatives (Shahwar *et al.*, 2009, 2011).

The molecular structure of the title compound is shown in Fig. 1. The essentially planar thiazole ring [r.m.s deviation 0.005 Å] forms dihedral angles of 16.85 (8)°  and 75.02 (8)° with the C5-C10 and C12-C17 phenyl rings, respectively. The dihedral angle between the two phenyl rings is 61.95 (9)°.

### Experimental

The title compound was prepared following a previously published method (Shahwar *et al.*, 2009). X-ray quality crystals were grown from a solution of the title compound in n-hexane:ethylacetate:methanol (6:3:1).

### Refinement

All H atoms were positioned with idealized geometry with C—H = 0.93 - 0.97 Å and were refined using a riding model with *U*_iso_(H) = 1.2 *U*_eq_(C). Four reflections 1 1 0, 0 0 1, 2 2 0 & 0 1 0 were omitted in the final refinemnt as they were obscured by the beamstop.

### Crystal data

**Table:**

C_17_H_13_NOS_2_	*Z* = 2
*M**_r_* = 311.40	*F*(000) = 324
Triclinic, *P*1	*D*_x_ = 1.396 Mg m^−^^3^
Hall symbol: -P 1	Mo *K*α radiation, λ = 0.71073 Å
*a* = 6.3152 (2) Å	Cell parameters from 6403 reflections
*b* = 10.8168 (3) Å	θ = 2.6–28.3°
*c* = 11.4545 (3) Å	µ = 0.36 mm^−^^1^
α = 84.1150 (9)°	*T* = 296 K
β = 77.6000 (9)°	Needle, pale yellow
γ = 76.1770 (9)°	0.35 × 0.31 × 0.15 mm
*V* = 740.99 (4) Å^3^	

### Data collection

**Table:**

Bruker KAPPA APEX II CCD diffractometer	3583 independent reflections
Radiation source: fine-focus sealed tube	2930 reflections with *I* > 2σ(*I*)
graphite	*R*_int_ = 0.028
φ and ω scans	θ_max_ = 28.4°, θ_min_ = 3.4°
Absorption correction: multi-scan (*SADABS*;Bruker, 2007)	*h* = −8→8
*T*_min_ = 0.886, *T*_max_ = 0.949	*k* = −14→14
13205 measured reflections	*l* = −15→15

### Refinement

**Table:**

Refinement on *F*^2^	Primary atom site location: structure-invariant direct methods
Least-squares matrix: full	Secondary atom site location: difference Fourier map
*R*[*F*^2^ > 2σ(*F*^2^)] = 0.036	Hydrogen site location: inferred from neighbouring sites
*wR*(*F*^2^) = 0.101	H-atom parameters constrained
*S* = 1.03	*w* = 1/[σ^2^(*F*_o_^2^) + (0.048*P*)^2^ + 0.1921*P*] where *P* = (*F*_o_^2^ + 2*F*_c_^2^)/3
3583 reflections	(Δ/σ)_max_ < 0.001
190 parameters	Δρ_max_ = 0.28 e Å^−^^3^
0 restraints	Δρ_min_ = −0.26 e Å^−^^3^

### Special details

**Table:**

Geometry. All s.u.'s (except the s.u. in the dihedral angle between two l.s. planes) are estimated using the full covariance matrix. The cell s.u.'s are taken into account individually in the estimation of s.u.'s in distances, angles and torsion angles; correlations between s.u.'s in cell parameters are only used when they are defined by crystal symmetry. An approximate (isotropic) treatment of cell s.u.'s is used for estimating s.u.'s involving l.s. planes.
Refinement. Refinement of *F*^2^ against ALL reflections. The weighted *R*-factor *wR* and goodness of fit *S* are based on *F*^2^, conventional *R*-factors *R* are based on *F*, with *F* set to zero for negative *F*^2^. The threshold expression of *F*^2^ > 2σ(*F*^2^) is used only for calculating *R*-factors(gt) *etc*. and is not relevant to the choice of reflections for refinement. *R*-factors based on *F*^2^ are statistically about twice as large as those based on *F*, and *R*- factors based on ALL data will be even larger.

### Fractional atomic coordinates and isotropic or equivalent isotropic displacement parameters (Å^2^)

**Table:**

	*x*	*y*	*z*	*U*_iso_*/*U*_eq_	
S1	0.36101 (6)	0.34929 (4)	0.98088 (4)	0.04416 (12)	
S2	0.24747 (8)	0.57799 (4)	0.82368 (4)	0.05631 (14)	
O1	−0.0562 (2)	0.18668 (12)	0.89696 (10)	0.0518 (3)	
N1	0.06938 (19)	0.37139 (12)	0.85045 (10)	0.0382 (3)	
C3	0.2282 (2)	0.22259 (14)	0.99118 (13)	0.0374 (3)	
C4	0.2515 (2)	0.11550 (14)	1.06168 (13)	0.0401 (3)	
H4	0.1636	0.0602	1.0540	0.048*	
C12	0.0019 (3)	0.36707 (14)	0.64596 (13)	0.0414 (3)	
C5	0.3954 (2)	0.07342 (14)	1.14880 (13)	0.0401 (3)	
C1	0.2109 (2)	0.43656 (15)	0.87728 (13)	0.0395 (3)	
C2	0.0659 (2)	0.25206 (14)	0.91071 (12)	0.0384 (3)	
C11	−0.0820 (2)	0.42216 (15)	0.76689 (13)	0.0430 (3)	
H11A	−0.1013	0.5141	0.7575	0.052*	
H11B	−0.2263	0.4036	0.8007	0.052*	
C6	0.5720 (3)	0.12723 (16)	1.15454 (15)	0.0481 (4)	
H6	0.6061	0.1920	1.0989	0.058*	
C10	0.3525 (3)	−0.02573 (16)	1.23169 (16)	0.0529 (4)	
H10	0.2383	−0.0650	1.2282	0.063*	
C7	0.6974 (3)	0.08547 (18)	1.24193 (17)	0.0554 (4)	
H7	0.8146	0.1226	1.2448	0.066*	
C13	−0.1276 (3)	0.30505 (19)	0.60087 (17)	0.0594 (5)	
H13	−0.2644	0.2959	0.6464	0.071*	
C17	0.2039 (3)	0.38009 (18)	0.57709 (15)	0.0541 (4)	
H17	0.2924	0.4226	0.6058	0.065*	
C8	0.6501 (3)	−0.01051 (19)	1.32454 (18)	0.0611 (5)	
H8	0.7336	−0.0378	1.3838	0.073*	
C9	0.4783 (3)	−0.06585 (19)	1.31873 (19)	0.0651 (5)	
H9	0.4466	−0.1312	1.3742	0.078*	
C16	0.2752 (4)	0.3298 (2)	0.46494 (17)	0.0692 (6)	
H16	0.4123	0.3378	0.4190	0.083*	
C14	−0.0563 (5)	0.2564 (2)	0.4888 (2)	0.0784 (6)	
H14	−0.1455	0.2152	0.4591	0.094*	
C15	0.1443 (4)	0.2685 (2)	0.42184 (18)	0.0751 (7)	
H15	0.1925	0.2351	0.3466	0.090*	

### Atomic displacement parameters (Å^2^)

**Table:**

	*U*^11^	*U*^22^	*U*^33^	*U*^12^	*U*^13^	*U*^23^
S1	0.0472 (2)	0.0448 (2)	0.0463 (2)	−0.01693 (16)	−0.01683 (16)	0.00314 (16)
S2	0.0700 (3)	0.0434 (2)	0.0624 (3)	−0.0228 (2)	−0.0220 (2)	0.00817 (19)
O1	0.0605 (7)	0.0539 (7)	0.0511 (7)	−0.0274 (5)	−0.0180 (5)	0.0010 (5)
N1	0.0415 (6)	0.0407 (7)	0.0344 (6)	−0.0112 (5)	−0.0093 (5)	−0.0016 (5)
C3	0.0404 (7)	0.0390 (7)	0.0334 (7)	−0.0110 (6)	−0.0054 (5)	−0.0041 (6)
C4	0.0456 (7)	0.0387 (7)	0.0369 (7)	−0.0114 (6)	−0.0061 (6)	−0.0053 (6)
C12	0.0501 (8)	0.0369 (7)	0.0364 (7)	−0.0052 (6)	−0.0152 (6)	0.0044 (6)
C5	0.0463 (7)	0.0344 (7)	0.0368 (7)	−0.0052 (6)	−0.0057 (6)	−0.0042 (6)
C1	0.0400 (7)	0.0418 (8)	0.0370 (7)	−0.0104 (6)	−0.0062 (6)	−0.0030 (6)
C2	0.0422 (7)	0.0427 (8)	0.0315 (7)	−0.0130 (6)	−0.0048 (5)	−0.0041 (6)
C11	0.0418 (7)	0.0464 (8)	0.0405 (8)	−0.0062 (6)	−0.0122 (6)	−0.0012 (6)
C6	0.0473 (8)	0.0483 (9)	0.0473 (9)	−0.0127 (7)	−0.0089 (7)	0.0082 (7)
C10	0.0631 (10)	0.0402 (8)	0.0598 (11)	−0.0171 (7)	−0.0197 (8)	0.0070 (7)
C7	0.0489 (9)	0.0575 (10)	0.0623 (11)	−0.0131 (7)	−0.0194 (8)	0.0050 (8)
C13	0.0733 (12)	0.0610 (11)	0.0518 (10)	−0.0236 (9)	−0.0208 (9)	−0.0012 (8)
C17	0.0549 (9)	0.0590 (11)	0.0459 (9)	−0.0099 (8)	−0.0092 (7)	0.0005 (8)
C8	0.0641 (11)	0.0582 (11)	0.0634 (11)	−0.0091 (8)	−0.0294 (9)	0.0113 (9)
C9	0.0786 (13)	0.0512 (10)	0.0684 (12)	−0.0191 (9)	−0.0279 (10)	0.0232 (9)
C16	0.0730 (12)	0.0707 (13)	0.0467 (10)	0.0048 (10)	−0.0005 (9)	0.0020 (9)
C14	0.1163 (19)	0.0720 (14)	0.0588 (13)	−0.0261 (13)	−0.0332 (13)	−0.0124 (10)
C15	0.1135 (19)	0.0592 (12)	0.0438 (10)	0.0038 (12)	−0.0195 (11)	−0.0085 (9)

### Geometric parameters (Å, °)

**Table:**

S1—C1	1.7390 (15)	C6—C7	1.382 (2)
S1—C3	1.7517 (15)	C6—H6	0.9300
S2—C1	1.6389 (16)	C10—C9	1.379 (2)
O1—C2	1.2066 (18)	C10—H10	0.9300
N1—C1	1.3614 (19)	C7—C8	1.374 (3)
N1—C2	1.4026 (19)	C7—H7	0.9300
N1—C11	1.4715 (18)	C13—C14	1.380 (3)
C3—C4	1.339 (2)	C13—H13	0.9300
C3—C2	1.480 (2)	C17—C16	1.388 (3)
C4—C5	1.459 (2)	C17—H17	0.9300
C4—H4	0.9300	C8—C9	1.374 (3)
C12—C13	1.378 (2)	C8—H8	0.9300
C12—C17	1.379 (2)	C9—H9	0.9300
C12—C11	1.503 (2)	C16—C15	1.368 (3)
C5—C6	1.392 (2)	C16—H16	0.9300
C5—C10	1.395 (2)	C14—C15	1.361 (3)
C11—H11A	0.9700	C14—H14	0.9300
C11—H11B	0.9700	C15—H15	0.9300
			
C1—S1—C3	92.81 (7)	C7—C6—H6	119.6
C1—N1—C2	116.64 (12)	C5—C6—H6	119.6
C1—N1—C11	123.42 (13)	C9—C10—C5	120.40 (16)
C2—N1—C11	119.89 (12)	C9—C10—H10	119.8
C4—C3—C2	121.65 (13)	C5—C10—H10	119.8
C4—C3—S1	128.94 (12)	C8—C7—C6	120.44 (17)
C2—C3—S1	109.35 (10)	C8—C7—H7	119.8
C3—C4—C5	129.10 (14)	C6—C7—H7	119.8
C3—C4—H4	115.5	C12—C13—C14	120.65 (19)
C5—C4—H4	115.5	C12—C13—H13	119.7
C13—C12—C17	118.90 (16)	C14—C13—H13	119.7
C13—C12—C11	119.66 (15)	C12—C17—C16	120.04 (18)
C17—C12—C11	121.41 (15)	C12—C17—H17	120.0
C6—C5—C10	118.12 (14)	C16—C17—H17	120.0
C6—C5—C4	123.75 (14)	C7—C8—C9	119.40 (16)
C10—C5—C4	118.12 (14)	C7—C8—H8	120.3
N1—C1—S2	127.66 (12)	C9—C8—H8	120.3
N1—C1—S1	111.06 (11)	C8—C9—C10	120.86 (17)
S2—C1—S1	121.28 (9)	C8—C9—H9	119.6
O1—C2—N1	122.92 (13)	C10—C9—H9	119.6
O1—C2—C3	126.97 (14)	C15—C16—C17	120.2 (2)
N1—C2—C3	110.11 (12)	C15—C16—H16	119.9
N1—C11—C12	112.72 (12)	C17—C16—H16	119.9
N1—C11—H11A	109.0	C15—C14—C13	120.1 (2)
C12—C11—H11A	109.0	C15—C14—H14	119.9
N1—C11—H11B	109.0	C13—C14—H14	119.9
C12—C11—H11B	109.0	C14—C15—C16	120.13 (19)
H11A—C11—H11B	107.8	C14—C15—H15	119.9
C7—C6—C5	120.75 (15)	C16—C15—H15	119.9
			
C1—S1—C3—C4	−176.86 (14)	C1—N1—C11—C12	101.92 (16)
C1—S1—C3—C2	0.27 (11)	C2—N1—C11—C12	−80.68 (17)
C2—C3—C4—C5	−177.53 (14)	C13—C12—C11—N1	122.62 (16)
S1—C3—C4—C5	−0.7 (2)	C17—C12—C11—N1	−59.2 (2)
C3—C4—C5—C6	−14.8 (2)	C10—C5—C6—C7	−1.6 (2)
C3—C4—C5—C10	164.69 (16)	C4—C5—C6—C7	177.88 (15)
C2—N1—C1—S2	−178.75 (11)	C6—C5—C10—C9	1.9 (3)
C11—N1—C1—S2	−1.3 (2)	C4—C5—C10—C9	−177.57 (16)
C2—N1—C1—S1	1.48 (16)	C5—C6—C7—C8	0.3 (3)
C11—N1—C1—S1	178.95 (10)	C17—C12—C13—C14	0.2 (3)
C3—S1—C1—N1	−0.97 (11)	C11—C12—C13—C14	178.45 (17)
C3—S1—C1—S2	179.24 (10)	C13—C12—C17—C16	−0.8 (3)
C1—N1—C2—O1	178.07 (14)	C11—C12—C17—C16	−179.01 (15)
C11—N1—C2—O1	0.5 (2)	C6—C7—C8—C9	0.8 (3)
C1—N1—C2—C3	−1.27 (17)	C7—C8—C9—C10	−0.4 (3)
C11—N1—C2—C3	−178.83 (12)	C5—C10—C9—C8	−1.0 (3)
C4—C3—C2—O1	−1.5 (2)	C12—C17—C16—C15	0.8 (3)
S1—C3—C2—O1	−178.84 (13)	C12—C13—C14—C15	0.4 (3)
C4—C3—C2—N1	177.84 (13)	C13—C14—C15—C16	−0.5 (3)
S1—C3—C2—N1	0.46 (14)	C17—C16—C15—C14	−0.1 (3)
